# Prevalence, Risk Factors, and Clinical Correlates of Insomnia in China College Student During the COVID-19

**DOI:** 10.3389/fpsyt.2021.694051

**Published:** 2021-08-06

**Authors:** Chang Yu, Xingxing Li, Gangqiao Qi, Liang Yang, Wenbo Fu, Qin Yao, Lei Wei, Dongsheng Zhou, Xiangyang Zhang, Hong Zheng

**Affiliations:** ^1^Ningbo Key Laboratory of Sleep Medicine, Ningbo Kangning Hospital, Ningbo, China; ^2^Taizhou Second People's Hospital, Taizhou, China; ^3^Shandong Medical College, Linyi, China; ^4^Heze Vocational College, Heze, China; ^5^Department of Respiratory, Zibo Central Hospital, Zibo, China; ^6^Department of Psychology, University of Chinese Academy of Sciences, Beijing, China

**Keywords:** insomnia, college students, COVID-19, mental health, AIS

## Abstract

**Objective:** The 2019 novel coronavirus disease (COVID-19) broke out in Hubei Province and spread rapidly to the whole country, causing huge public health problems. College students are a special group, and there is no survey on insomnia among college students. The purpose of this study was to investigate the incidence and related factors of insomnia in college students during the period of COVID-19.

**Method:** A total of 1,086 college students conducted a cross-sectional study through the questionnaire star platform. The survey time was from February 15 to February 22, 2020. The collected information included demographic informatics and mental health scale, Athens Insomnia Scale (AIS) to assess sleep quality, Self-Reporting Questionnaire-20 (SRQ-20) to assess general psychological symptoms, Chinese perceived stress scale (CPSS) to assess stress. We used logistic regression to analyze the correlation between related factors and insomnia symptoms.

**Results:** The prevalence of insomnia, general psychological symptoms and stress were 16.67, 5.8, and 40.70%, respectively. Multivariate logistic regression analysis showed that gender (*OR* = 1.55, *p* = 0.044, 95% *CI* = 1.00–2.41), general psychological symptoms (*OR* = 1.49, *p* < 0.01, 95% *CI* = 1.40–1.60) and living in an isolation unit (*OR* = 2.21, *p* = 0.014, 95% *CI* = 1.17–4.16) were risk factors for insomnia of college students.

**Conclusion:** Our results show that the insomnia is very common among college students during the outbreak of covid-19, and the related factors include gender, general psychological symptoms and isolation environment. It is necessary to intervene the insomnia of college students and warrants attention for mental well-being of college students.

## Introduction

The coronavirus disease 2019 (COVID-19) was broke out began at the end of 2019 and had spread rapidly across the whole country due to its rapid spread, meanwhile, the number of confirmed cases in the world is also increasing, Not only has it caused great public concern and panic (1), it has also brought huge psychological distress, including anxiety, depression, insomnia and other psychological problems, which can lead to suicide in serious cases. The Chinese government introduced the “I stay home” decree, which ordered people to stay at home which can lead to depression, anxiety, insomnia, and stress related to outbursts of uncertainty (2) that seriously afflicted people's mental health.

As a result of the epidemic, universities have post-pone the reopening of schools and students have to study progress by taking online courses at home, also graduates can't find jobs in time. They are more confused about the future, which may cause mental health problems such as anxiety, depression, fear and insomnia.

At present, people pay more attention to depression and anxiety and there is less investigation on insomnia. However, insomnia during the epidemic period has clinical and prognostic significance, because it may bring a lot of negative effects on daily life, long-term insomnia will develop into depression and anxiety, which has been proved by previous experiments (3–5) and further research shows that people who meet the standard of insomnia are more likely to have stress, fatigue and mental health problems, which lead to the decline of quality of life and high risk of neurological diseases (6, 7). However, as far as we know, the current researches on insomnia during the COVID-19 are all aimed at medical staff. Zhang's team found that the insomnia rate of medical staff in Wuhan is as high as 38.4%, far higher than that of the general population, which is consistent with that of Hu's team (34.0%) (8, 9). However, the epidemiological data and risk factors of insomnia in college students are not clear.

In this study, we investigated the prevalence of insomnia symptoms and the risk factors of insomnia in college student, so as to provide appropriate management strategies practical, targeted interventions to improve the symptoms of insomnia.

## Materials and Methods

### Study Design

The study was approved by the Clinical Research Ethics Committee of Ningbo Kangning Hospital. The questionnaire was designed by epidemiologists. All the questionnaires were collected online through the questionnaire star platform (https://www.wjx.cn/jq/65379393.aspx). From February 15 to February 22, a total of 1086 questionnaires were collected in this cross-sectional study. The subjects came from college students from multiple provinces of china. In order to ensure repeated filling of the questionnaire, the same IP address can only be submitted once. The demographic includes age, gender, education, occupation, etc. the Athens Insomnia (AIS), Chinese perceived stress scale (CPSS) and Self-Reporting Questionnaire-20 (SRQ-20) were administered to screen insomnia (i.e., ≥6 in AIS), stress (i.e., ≥29 in CPSS), and the general psychological symptoms (i.e., ≥7 in SRQ-20), separately. The survey is anonymous and the participants can give up at any time. The information in the questionnaire ensures confidentiality. All subjects provided informed consent to participate in the study.

### Measurement Tools

Athens Insomnia Scale (AIS), an internationally recognized eight-item self-assessment scale used to assess the quality of sleep. Each item of the scale is divided into 0, 1, 2, and 3 grades from nothing to seriousness. An AIS total score ≥6 indicates that insomnia is present, whereas a total score of <6 indicated no sleep disorders (10).

Chinese Perceived Stress Scale(CPSS): Yang Tingzhong revised the Chinese version based on the perceptual pressure scale (PSS) compiled by American scholar Cohen et al. (11) In 1983. The purpose is to detect the overall and prevalent pressure in the life of the crowd. Each entry has 5 grades ranging from 0 = “never” to 4 = “very often. The scale can be divided into negative subscale (items 1, 2, 3, 8, 11, 12, and 14) and positive subscale (item 4, 5, 6, 7, 9, 10, and 13. The higher the total score, the more stress the patient feels. The total score is ≥29, indicating the presence of stress (12).

The Self-report Questionnaire (SRQ-20) was used to assess common mental disorders such as anxiety and depression, which consists of 20 “yes/no” questions, including four about physical symptoms and 16 about mental and emotional disorders. Responses were scored using “0” or “1,” where “1” indicates the presence of symptoms in the past month, and “0” indicates the absence of symptoms. The clinical reference index of SRQ-20 is 7, where a score exceeding 7 is considered to reflect emotional pain (13).

### Statistical Analysis

The data were analyzed with spss23.0 for Windows, All demographic and clinical data, including insomnia scores, were normally distributed among the investigators, and were expressed by descriptive statistics (n, %). Demographic information and clinical variables were tested by chi square test. The incidence of insomnia was described by percentage. The factors related to insomnia were analyzed by binary logistic regression. The correlation between insomnia and demographic and clinical variables was detected by Pearson correlation. Stepwise multiple regression analysis was then used to identify predictors associated with insomnia. All tests were two-tailed with the significance level set at 0.05.

## Results

A total of 1,086 college students participated in the survey. The prevalence of insomnia, anxiety and depression physical symptoms and stress-related symptoms among students are 16.67% (AIS ≥ 6), 5.80% (SRQ-20 ≥ 8) and 40.70% (CPSS ≥ 29), respectively.

Table 1 shows the demographic data and scale information of insomnia and non-insomnia in students. The report shows that the majority of insomnia students are female (75.14%), and there are significant differences in gender (*x*^2^ = 5.27, *P* = 0.02) compared with non-insomnia, and they have higher anxiety and depression physical symptoms (25.41 vs. 1.88%, *x*^2^ = 152.9, *P* < 0.01) and stress symptoms (54.70 vs. 37.90%, *x*^2^ = 17.63, *P* < 0.01). In addition, whether in an isolation unit (97.79 vs. 92.93%, *x*^2^ = 6.07, *P* = 0.014) was also a risk factor for insomnia.

**Table 1:** Basic demographic and clinical characteristics of college students.

	**Insomnia (*n* = 181)**	**Non-insomnia (*n* = 905)**	***F*/χ^2^**	***P***
Gender, % (n)			5.27	0.02
female	75.14 (136)	66.41 (601)		
Male	24.86 (45)	33.59 (304)		
Age, % (n)			2.28	0.13
<20 years	39.78 (72)	33.92 (307)		
20–40 years	60.22 (109)	66.08 (598)		
41–60 years	0 (0)	0 (0)		
>60 years	0 (0)	0 (0)		
Marital status, % (n)			0.40	0.53
Married/cohabitating	1.10 (2)	0.66 (6)		
Other	99.90 (179)	99.34 (899)		
Anxiety/depression (SRQ total score), % (n)			152.90	<0.01
Yes (>7)	25.41 (46)	1.88 (17)		
No (0–7)	74.59 (135)	98.12 (888)		
Pressure (CPSS total score), % (n)			17.63	<0.01
Yes (>29)	54.70 (99)	37.90 (343)		
No (0–29)	45.30 (82)	62.10 (562)		
Whether have confirmed crown patient or not in Community, % (n)			2.27	0.132
Yes	9.39 (17)	6.30 (57)		
No	90.61 (164)	93.7 (848)		
Whether to be quarantined, % (n)			6.07	0.014
Yes	97.79 (177)	92.93 (841)		
No	2.21 (4)	7.07 (64)		

Binary logistic regression analysis showed that gender (*OR* = 1.55, *p* = 0.044, 95% *CI* = 1.00–2.41), general psychological symptoms (*OR* = 1.49, *p* < 0.01, 95% *CI* = 1.40–1.60), and whether living in an isolation unit (*OR* = 2.21, *p* = 0.014, 95% *CI* = 1.17–4.16) were closely related to insomnia in students. Pearson correlation analysis showed that Insomnia Scale score was significantly correlated with gender (*r* = 0.43, *P* = 0.04), general psychological symptoms (*r* = 0.63, *P* < 0.01), stress (*r* = 0.23, *P* < 0.01), and isolation (*r* = 0.64, *P* < 0.01). Further linear regression showed that general psychological symptoms (beta = 0.6, *t* = 24.44, *P* < 0.01), isolation (beta = 0.089, *t* = 3.67, *P* < 0.01) were still related to Insomnia.

## Discussion

As far as we know, this is the first survey on the incidence of insomnia and related factors among college students during the outbreak of covid-19. We found that the insomnia is very common among college students during the outbreak of covid-19. In addition, some demographic and clinical variables are also considered as risk factors for insomnia college students, such as gender, stress, physical symptoms and Isolation or not.

We found that the insomnia rate of college students in the epidemic was 16.67%. Interestingly, in a previous study, 9.5% of united college students met proposed DSM-5 criteria for chronic insomnia (14). In another more rigorous epidemiological study, Lichstein et found that the prevalence of insomnia in young people aged 20–29 years was similar to 9.1% (15). In general, the current research shows that the prevalence of insomnia in college students is far greater than the previous studies. Insomnia is also a very common disease in the general population. Due to the different definitions of insomnia, the prevalence varies from 5 to 30%. Generally speaking, about 10% of insomnia patients are clinically recognized (16, 17), so the rate of insomnia in the general population was also higher than past.

The epidemic will bring huge pressure. There are literature reports that the relationship between stress and sleep is interact (18), during the outbreak of SARS, a survey found that the sleep quality of medical workers was the worst during the crisis (19). With the alleviation of the epidemic, the psychological pressure decreased, and the sleep quality gradually improved, indicating that insomnia is related to the pressure caused by the epidemic (20). This study also verified this view, because of the pressure brought by the covid-19 epidemic, leading to different groups of insomnia rate has significantly increased.

Our survey found that the insomnia of college students in the epidemic was related to gender, and women were more likely to have insomnia, which was consistent with the results of another study in Italy (21). This may be related to hormones. The change of hormones after puberty is considered to be a factor leading to gender differences in adult sleep. In women, the level of sexual steroids is characterized by menstrual cycle. During the whole menstrual period, various hormone levels are related to sleep latency, sleep efficiency and sleep quality (22, 23). Some studies have shown that the peak values of estradiol and progesterone are related to the increase of arousal times and the prolongation of awake time (24). During the period of covid-19, a survey on sleep of medical staff also found that women were more likely to suffer from insomnia (25). In addition, another survey on sexual intercourse during SARS found that insomnia was more common among women (19). These results are consistent with ours.

Our report found that insomnia students are more likely to have anxiety, depression, somatization disorders and other general psychological symptoms. It may be that college students are more likely to get all kinds of negative news about covid-19 due to their frequent use of mobile phones, which makes them more sensitive to self-health and the spread of the virus. Moreover, due to the increasing number of suspected cases and confirmed cases, the fear of self-safety is increased. In addition, due to the shortage of materials such as masks, people's fear is further increased (26). All of these increase the pressure invisibly, which is physical and psychological. It may also activate HPA and lead to sleep disorder. Furthermore, sleep disorders may lead to further activation of HPA, leading to a vicious cycle of insomnia and stress (27).

In addition, during the outbreak of covid-19, in order to respond to the call of the country, everyone needs to isolate themselves at home, which makes people feel lonely, bored, scared and even angry. In a survey of medical workers, it was found that the insomnia rate of people working in isolated environment was 1.71 times higher than that of ordinary people (28). And because the epidemic situation is not well controlled, leading to the increase of isolation time, the longer the isolation time, indicating more negative results, the more prone to anger and avoidance behavior, greater psychological pressure, leading to more serious insomnia (29).

This study has some limitations. First, the sample size is still relatively small. This is a cross-sectional study, unable to explore the relationship between insomnia and other variables. More longitudinal studies are needed. Secondly, the incidence of insomnia in college students before the epidemic is lack of investigation, so there is no suitable control to match. Third, this is a research based on the scale. The self-assessment scale for insomnia could not be used for the diagnosis of insomnia. Moreover, through the survey of online questionnaire, we hope there will be more face-to-face clinical interviews in the future. Fourth, it was not clear how many subjects received links to the survey, so participation rates could not be assessed. and we also did not investigate whether existing pathologies or the use of pharmacological therapies which can affect sleep quality. And the questionnaire is self-designed, there is no standard questionnaire to investigate social and psychological factors. Finally, the data in this article comes from college students and cannot be extended to other populations at present.

In conclusion, during the period of covid-19, the prevalence of insomnia in students is much higher than usual. We should not only pay attention to the patients, but also to the college student groups. For those with problems, we should give timely targeted treatment, including Cognitive insomnia behavior therapy (CBTI) (30) to understand the problems of insomnia in college students, which is conducive to effective mental health education and training.

## Data Availability Statement

The raw data supporting the conclusions of this article will be made available by the authors, without undue reservation.

## Ethics Statement

The study was approved by the Clinical Research Ethics Committee of Ningbo Kangning Hospital. All subjects provided informed consent to participate in the study.

## Author Contributions

HZ, DZ, and LW designed the study. CY, XL, GQ, WF, and DZ collected the survey data. CY, XL, DZ, LW, and HZ analyzed the results and wrote the manuscript. All authors have read and approved the final version of the manuscript.

## Conflict of Interest

The authors declare that the research was conducted in the absence of any commercial or financial relationships that could be construed as a potential conflict of interest.

## Publisher's Note

All claims expressed in this article are solely those of the authors and do not necessarily represent those of their affiliated organizations, or those of the publisher, the editors and the reviewers. Any product that may be evaluated in this article, or claim that may be made by its manufacturer, is not guaranteed or endorsed by the publisher.
